# Physicochemical properties, nutritional value, and antioxidant potential of jackfruit (*Artocarpus heterophyllus*) pulp and seeds from Cameroon eastern forests

**DOI:** 10.1002/fsn3.3437

**Published:** 2023-05-22

**Authors:** Ulrich Landry Kamdem Bemmo, Jean Marcel Bindzi, Pamela Regine Tayou Kamseu, Serge Cyrille Houketchang Ndomou, Stephano Tene Tambo, François Ngoufack Zambou

**Affiliations:** ^1^ Department of Life Science, Higher Teacher Training College University of Bertoua Bertoua Cameroon; ^2^ Research Unit of Biochemistry, Food Science and Nutrition (URBPMAN), Department of Biochemistry, Faculty of Science University of Dschang Dschang Cameroon; ^3^ CRESA Forêt‐Bois, Faculty of Agronomy and Agricultural Science University of Dschang Yaounde Cameroon

**Keywords:** antioxidant potential, jackfruit, nutritional value, pulp, seeds

## Abstract

This work aimed to study the physicochemical, nutritional, and antioxidant characteristics of the pulp and seeds of the jackfruit collected in the East Cameroon region to reduce the malnutrition problems encountered in the said region. To achieve this work, we first administered a survey. We observed that 50 people from Bertoua knew the fruit among the 200 people surveyed. After that, the physicochemical characteristics and nutritional value of *Artocarpus heterophyllus* pulp and seeds from Bertoua were evaluated. Finally, the antioxidant compounds (total phenolic and flavonoid contents) and the antioxidant activities were evaluated through the DPPH free radical scavenging capacity, the ability to reduce ferric ions, and the hydroxyl radical scavenging ability using the aqueous, ethanolic, and hydro‐ethanolic extracts of these samples (pulp and seeds). Results (means ± standard deviation) were obtained in triplicate and analyzed statistically by the analysis of variance (ANOVA) at the 0.05 probability level. The results revealed that the jackfruit pulp has a water content (89.85% ± 0.49) significantly higher than that of seeds (60.075% ± 0.12). The pH of jackfruit pulp (5 ± 0) is significantly lower than that of seeds (6 ± 0). Jackfruit pulp had a carbohydrate content (54.39% ± 0.47) significantly higher than that of seeds (49.01% ± 0.43). The protein content of jackfruit pulp (18.35% ± 0.04) is lower than that of the seeds (21.66% ± 0.31). For mineral content, the maximum contents of K (848 mg/100 g ± 10.34) and Na (69.53 mg/100 g ± 0) were identified in the jackfruit pulp while those of Ca (132 mg/100 g ± 9.42), Mg (43.73 mg/100 g ± 9.12), and P (101.51 mg/100 g ± 4.02) were found in the seeds. However, all extracts possessed both antioxidant compounds (phenols and flavonoids) and antioxidant activities. In conclusion, the jackfruit appears to be a fruit that can improve the nutritional status of the populations of eastern Cameroon.

## Graphical abstract

This is the first article on the pulp and seeds of Jackfruit in Cameroon. This article presents the nutritional value and antioxidant potential of the pulp and seeds of Jackfruit harvested in Bertoua, Cameroon.

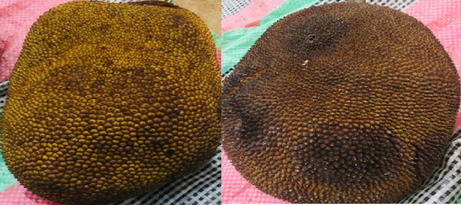

## 1 INTRODUCTION

In Cameroon, as in most developing countries, the issue of malnutrition remains a challenge. Malnutrition is defined as deficiencies, excesses, or imbalances in a person's energy and/or nutritional intake. In Cameroon, 33% of children under the age of five suffer from chronic malnutrition (INS, 2020). In the East Cameroon region, 37% of children suffer from stunting and 65% of children aged 6–59 months have anemia (INS, 2020). The management of malnutrition is done through hospitalizations in nutritional centers. However, it is not effective because of the lack of competent personnel, the high cost of care, the inaccessibility of care in remote areas, or the shortage of therapeutic foods (Nguefack et al., 2015). One possibility to reduce this malnutrition is the valorization of local food products and more specifically forest fruits because their nutritional potential and their almost free availability make them accessible to all social classes (Kone, 2018).

The Eastern Region is one of the largest regions in Cameroon and is an ecological area dominated by large trees and a diversity of plant species (Mengue, 2004); many fruits are little known and consumed by the population. They are generally underexploited but could constitute a significant source of nutrients. Among these fruits, we find the fruit of *Artocarpus heterophyllus* commonly called jackfruit, which is the largest known edible fruit. Studies conducted in India and Bangladesh have shown that jackfruit is a fruit rich in water and carbohydrates; however, its fat content is low. It contains micronutrients such as vitamins (thiamine, riboflavin, vitamin C, and vitamin A) and minerals (potassium, sodium, calcium, magnesium, and iron) necessary for the proper functioning of the human body (Goswami & Chacrabati, 2016; Haq, 2006). Jackfruit seeds are less widely known but have important nutritional value. Jackfruit seed contains moisture 21.10%–71.92%; fiber 0.94%–3.96%; ash 0.89%–3.16%; protein 10.09%–18.12%; and carbohydrate content 7.89%–30.84% (Kushwaha et al., 2023; Thatsanasuwan et al., 2023). Jackfruit seeds are used as a component of food formulations including gluten‐free pasta made from jackfruit seeds (Thatsanasuwan et al., 2023), jackfruit seed flour‐based waffle ice cream cone (Kushwaha et al., 2023), and jackfruit seed starch (Kaur et al., 2023).

In Cameroon, there is no information related to this fruit; the nutritional values of a plant are a function of pedological and climatic factors, this study aimed to determine the physicochemical characteristics, the nutritional value, and the antioxidant properties of the pulp and seeds of the fruit *A. heterophyllus* to valorize it on a national scale and to contribute to improving the nutritional status of the populations of the East region in particular and of Cameroon in general.

## 2 MATERIALS AND METHODS

### 2.1 Knowledge and consumption surveys

**FIGURE 1:**
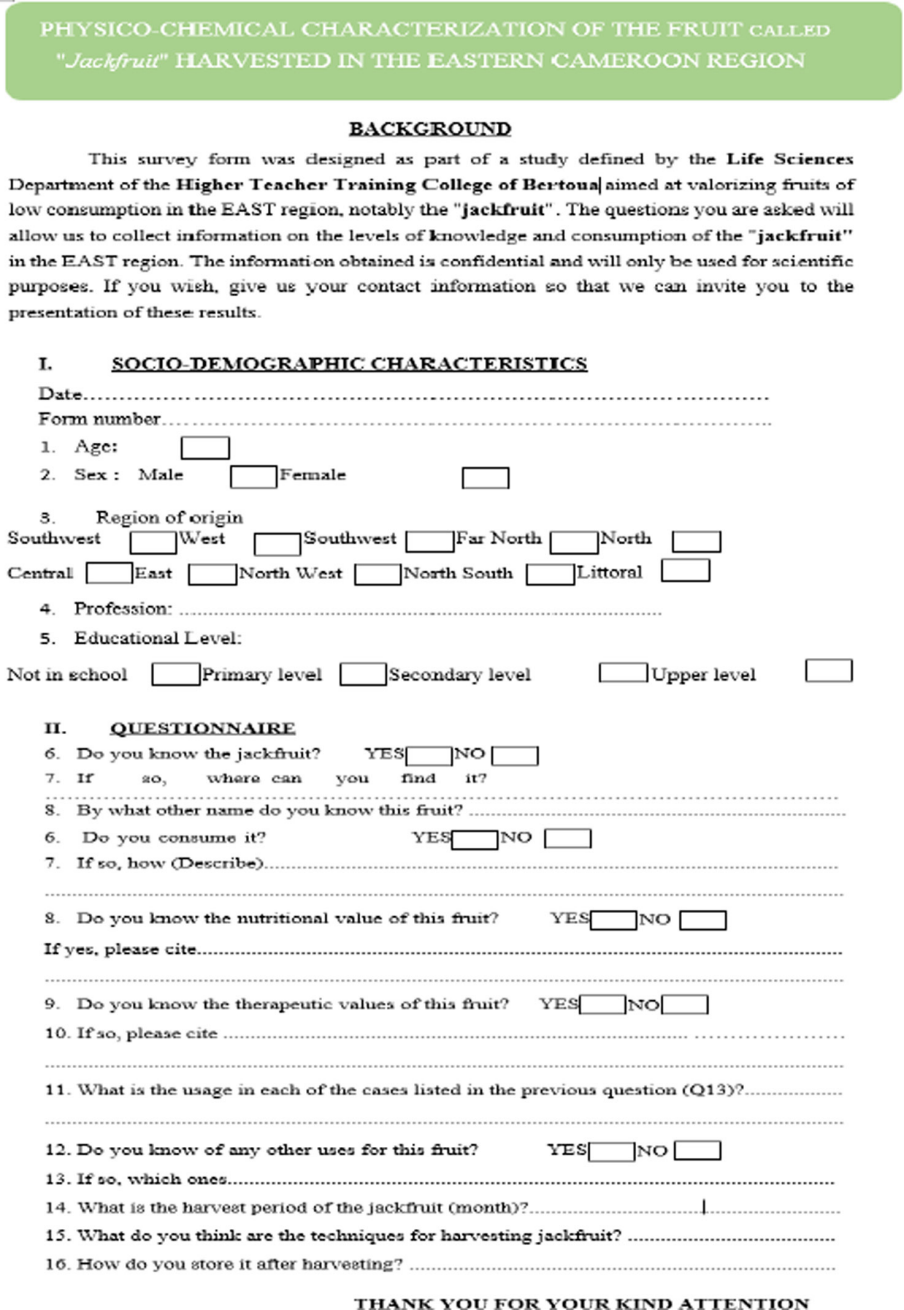
Consumption and knowledge survey form at the jackfruit.

A survey of knowledge and consumption of jackfruit was conducted in the city of Bertoua in the East Cameroon region. Approximately 200 people were randomly selected to be interviewed, but only 50 participated in the study. The criterion for inclusion in the study was knowledge of the fruit. It should be noted that the questionnaire developed was tested and then amended before printing the final version (Figure 1). It was then administered using a participatory approach. The questionnaire was designed as shown by Bemmo et al. (2023) to provide information on:

- Levels of knowledge of *A. heterophyllus* fruit;
- Socio‐cultural characteristics of respondents (age, gender, region of origin, educational level, and occupation);
- Modes of consumption of the fruit of *A. heterophyllus*;
- Common names of the fruit of *A. heterophyllus*;
- Nutritional (fruit) and therapeutic qualities of *A. heterophyllus*;
- Periods and techniques of harvesting the fruit of *A. heterophyllus*.

### 2.2 Collection and preparation of samples

**FIGURE 2:**
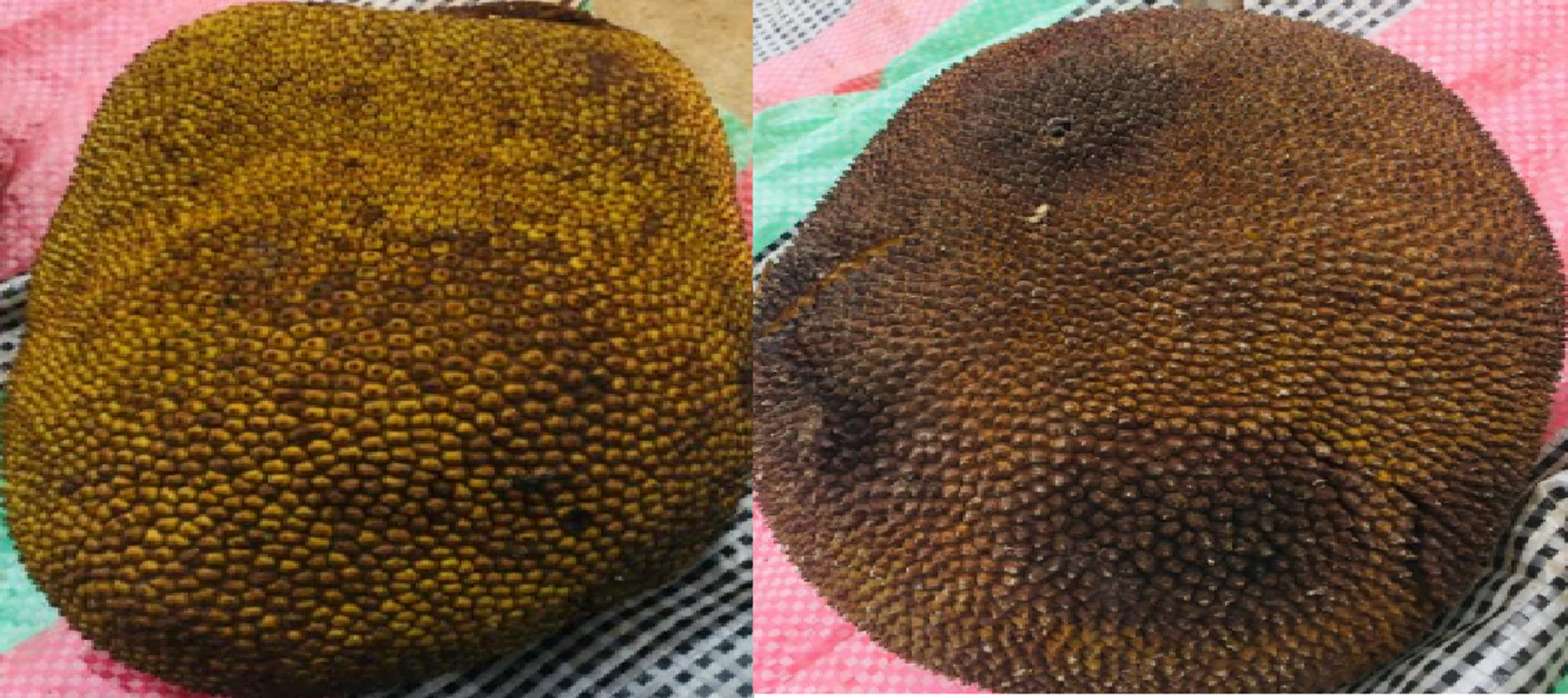
Photograph of the fruits of *Artocarpus heterophyllus* harvested.

**FIGURE 3:**
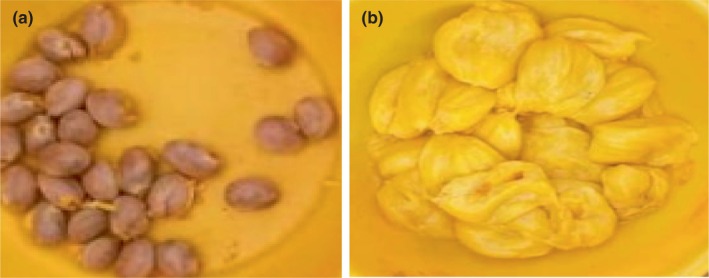
(a) Photograph of Jackfruit seeds and (b) Photograph of Jackfruit pulp.

The plant material consisted of ripe fruits of *A. heterophyllus* (Figure 2) harvested in the East Cameroon region, more precisely in the Mokolo III district (4°58′North and 13°40′East) of the city of Bertoua, the Bertoua II district and the Lom and Djerem department in July 2021. After harvesting, the fruits were sorted, cleaned (washed with soap and water), and then the pulp and seeds were extracted (Figure 3). The seeds were then boiled for 20 min, and the pulp and boiled seeds were dried in an oven (MEMERT) at a temperature of 45°C. Finally, they were crushed.

### 2.3 Physicochemical properties

The moisture content was determined according to the method of AOAC (1990). The ash content was evaluated by incineration of the sample for 20 h at 550°C according to the method of AOAC (1990). The determination of pH was made with a digital pH meter (HANNA HI 8424; Sadler & Murphy, 2010). The titratable acidity was determined in milliequivalent of NaOH per 100 g of dried matter (Sadler & Murphy, 2010). All analyses were carried out in triplicate.

### 2.4 Nutritional value

For the determination of the protein content, Nitrogen (N) content was analyzed using the Kjeldahl method, and the protein content was calculated as N × 6.25. Lipid content (mg/100 g DM) was determined by using the Soxhlet method (Ijarotimi & Keshinro, 2013). The carbohydrate content was determined by different methods as reported by Ijarotimi and Keshinro (2013). The fiber content was determined by the AOAC method (1990). All analyses were carried out in triplicate.

The energy value of the fruits (in Kcal/100 g) was determined by the method of Ijarotimi and Keshinro (2013). First, the contents of dry matter, total protein, total fat (lipids), and ash were determined, then the carbohydrate content was calculated by difference, and finally the energy value was calculated by multiplying the contents of each component (in g) by the average caloric values (in Kcal) and sum. The energy value was determined by Equation 1:

(1) $$W\left(\text{Kcal}\right)=9L+4P+4C$$

where, L is the lipid content; P is the protein content; C is thehydrolyzable carbohydrate content; and L, P, and C are expressed as a percentage of dry matter.

### 2.5 Determination of mineral content: Ca, Mg, K, Na, and P

The minerals K, Ca, Mg, and Na were determined by atomic absorption spectrophotometry following the method of AOAC (2005). Phosphorus was determined by the colorimetric method described by AOAC (2005).

### 2.6 Antinutrient content

Phytate content, Oxalate content, and Saponin content were determined using the standard method of AOAC (2005).

The tannins contents (condensed tannins and hydrolyzable tannins) were evaluated as follows:

Extraction of tannins: In a 50‐mL Erlenmeyer, we introduced 2 g of samples and 30 mL of 80% acetone. After 15 min of agitation, the mixture was filtered, the residue washed twice, and the acetone has been separated from the filtrate using a Rotavapor at 35°C.

Determination of condensed tannin content: Condensed tannins were determined by following the method of Kassegn (2018). Extracts were diluted to 20th before determination. The assay is performed in 15‐mL test tubes previously covered with aluminum foil. One milliliter of diluted sample was introduced into each test tube followed by 3 mL of a freshly prepared solution of 4% vanillin in ethanol (w/v). The mixture was then shaken and 1 mL of concentrated HCl was added. The tubes were left at room temperature for 15 min and then the absorbance was read at 500 nm. The equation below was used to determine the absorbance of 100 g of dry matter in the delipidated samples.

(2) $$Q=\left[\frac{F\times q\times 100}{\left(\mathrm{MS}\times m\right)}\right]\times 100$$

where, *F* is the dilution factor; *q* is the number of tannins from each trial; DM is the dry matter content of samples analyzed; and *m* is the mass of the sample used for the extraction.

Determination of hydrolyzable tannin content: Hydrolyzable tannins (mg/100 g DM) were determined by the method of Dif et al. (2015). One milliliter of the extract was added to 3.5 mL of a prepared solution of 0.01 M ferric trichloride (FeCl_3_) and 0.001 M chloride acid (HCl). The absorbance of the mixture was read 15 s after the addition of the reagent at a wavelength of 660 nm on a Ultraviolet spectrophotometer. The results were expressed using Equation (3):

(3) $$\mathrm{TH}=\left(\mathrm{Abs}\times M\times V\right)/E\;\text{mole}\times P$$

where, TH is the hydrolyzable tannins; Abs is the Absorbance; *M* is the mass = 300; *V* is the volume of the extract used; E mole: 2169 (constant expressed in moles) *P* is the weight of the sample.

### 2.7 Evaluation of antioxidant content

#### 2.7.1 Determination of total phenolic content

The total phenolic content of jackfruit samples was determined using the Folin–Ciocalteu colorimetric method, as described by Kassegn (2018). In a test tube of 5 mL volume, 0.01 mL of a 5 mg/mL extract solution was added, followed by the Folin–Ciocalteu reagent (0.2 mL) and distilled water (1.39 mL). After 3‐min incubation of the solution mixture at room temperature, 0.4 mL of 20% sodium carbonate solution was added and the mixture was re‐incubated for 20 min under the same conditions. The absorbance of the resulting blue‐colored solution was measured at 760 nm using a spectrophotometer (BioMate). The total phenolic content of the extract was calculated from the gallic acid standard curve and expressed as milligrams of gallic acid equivalent (GAE) per gram of extract.

#### 2.7.2 Determination of flavonoid content

Flavonoid content was determined according to the method described by Jahromi (2019). Approximately 0.1 mL of extract was mixed with 1.4 mL of distilled water before the introduction of 0.03 mL of 5% sodium nitrite (NaNO2) solution. After 5 min, 0.2 mL of 10% aluminum trichloride (AlCl3) solution was added. After resting for 5 min, 0.2 mL of 10% (NaOH) solution and 0.24 mL of distilled water were added and the absorbance was measured at 510 nm using a spectrophotometer (BioMate). The flavonoid content was determined using a standard curve obtained with catechin. The contents were expressed as milligrams catechin equivalent (CE) per gram of extract.

### 2.8 Evaluation of antioxidant activities

The antioxidant activity was evaluated by three tests: the 2,2‐diphenyl‐1picrylhydrazyl radical (DPPH) free radical scavenging capacity, hydroxyl radical scavenging ability, and the ability to reduce ferric ion (FRAP test).

#### 2.8.1 Determination of the antioxidant activity by the DPPH free radical scavenging assay

The ability of each plant extract to scavenge the DPPH free radical was determined according to the method of Mensor et al. (2001). A total volume of 4.5 mL of 0.002% alcoholic solution of DPPH was added to 0.5 mL of different concentrations (200; 100; 50; 25; 12.5; and 6.25 μg/mL) of extract samples and standard solutions (vitamin C) separately, to have final concentrations of products of 25–200 μg/mL. The samples were kept at room temperature in the dark for 30 min. The absorbance of the resulting solution was measured at 517 nm using a spectrophotometer (BioMate). The absorbance of the samples, control, and blank was measured in comparison with distilled water. The antioxidant activity (AA) was calculated as follows:

$$\mathrm{AA}\%=\left[\frac{\left({\mathrm{Abs}}_{\text{control}}-{\mathrm{Abs}}_{\text{sample}}\right)\times 100}{{\mathrm{Abs}}_{\text{control}}}\right]$$

Besides, the efficient concentration 50 (EC50) was determined according to the antioxidant activity.

##### Measurement of hydroxyl radical inhibition power (ROH)

The scavenging activity for hydroxyl radical produced by the Fenton reaction was measured using the orthophenanthroline method (Nagulendran et al., 2007). In this experiment, 60 μL of FeCl2 (1 mM), 90 μL of 1,10‐phenanthroline (1 mM), 2.4 mL of buffer 0.2 M phosphate (pH 7.8), 150 μL of 0.17 M hydrogen peroxide, and 1.5 mL of extracts prepared at different concentrations (200; 100; 50; 25; and 12.5 μg/mL) were mixed in the test tubes. The reaction was initiated with hydrogen peroxide and then incubated at room temperature for 5 min. After incubation, the absorbance of the mixture was recorded at 560 nm using the spectrophotometer (BioMate) against the blank prepared in the same conditions with distilled water. BHT solution was used as standard, and experiments were performed in triplicate. An increase in the absorbance of the reaction mixture indicated an increase in the ability to reduce the hydroxyl radical.

##### Assessment of the ferric reducing antioxidant power (FRAP)

The FRAP assay was determined by the method of Rechek et al. (2021), in test tubes previously containing 0.5 mL of extract solution at concentrations: 2000; 1000; 500; 250; and 125 μg/mL, prepared in methanol, 1 mL of potassium phosphate buffer solution (0.2 M, pH 6.6) and 1 mL of 1% aqueous potassium hexacyanoferrate solution [K3Fe (CN) 6] was added. The whole mixture was incubated for 30 min at 50°C in a water bath and then 1 mL of a 10% trichloroacetic acid solution was added. The mixture was centrifuged for 10 min and then, 1.5 mL of supernatant was taken and mixed with 1.5 mL of distilled water, followed by 0.1 mL of 0.1% FeCl_3_ ethanolic solution. A standard metal reductant, catechin, was prepared under the same conditions to compare the reducing power of the extracts. The blank consisted of all reagents except the extract. The absorbance of the reaction mixture was read at 700 nm against this blank on a HELIOS Epsilone spectrophotometer. An increase in absorbance of the reaction mixture indicated an increase in reducing power.

### 2.9 Statistical analysis

Results (means ± standard deviation) were obtained in triplicate and analyzed statistically by the analysis of variance (ANOVA) at the 0.05 probability level. Fisher tests were used to compare means of physicochemical, nutritional, and antioxidant evaluations using the factor program of the XLSTAT 2007 statistical package.

## 3 RESULTS

### 3.1 Knowledge and consumption surveys

Fifty people from the city of Bertoua took part in the study. This panel was made up of women (56%) and men (44%) distributed as follows: 92% from the East, 4% from the North‐West, 2% from the South, and 2% from the South‐West. The most represented age groups were those between 20 and 40 years old (56%). Of these, 50% had at least secondary education. Most of them were shopkeepers (38%) and students (28%). Regarding knowledge of the fruit, only 25 (25%) out of 200 randomly selected people knew the jackfruit. Regarding the location and common names of the fruit, out of 50 people who took part in the study, 72.7% thought that the jackfruit was found in the forest, 20% thought that it was found in front of houses, and 7.3% in swampy areas; almost half of the study population knew the fruit by the name of jackfruit (44.4%), 18.5% by the name of moom, and only 3.7% by the name of jackfruit.

In terms of consumption, only 24.5% consumed it, out of 50 people who took part in the study, 98% consumed jackfruit, and only 2% did not. Jackfruit was consumed in various forms in the East Cameroon region. Seventy‐three percent of people consumed the pulp of the Jackfruit after it had ripened completely, 18% consumed the boiled or burned seeds, and only 7.5% consumed it in processed form (6% prepared juices and 1.5% prepared dishes based on jackfruit). Apart from food, 48% of the study population knew of other uses for the *A. heterophyllus* plant; 38.6% knew that its wood is used for heating, 8.8% knew that its wood is used as formwork, but only 1.8% knew of its use in branding.

Regarding nutritional values, 96% of those who took part in the study knew the nutritional value of jackfruit. Half of them were aware of its high sugar content, but only 11.6% were aware of its high moisture content. At the same time, 68% of the study population knew about the therapeutic values of *A. heterophyllus*; among them, 25.5% knew that the peels treat many diseases, 3.6% knew about the fruit's ability to fight constipation, and only 1.8% were aware that the Jacque juice is used to soothe.

Regarding the different uses and harvesting periods of jackfruit, apart from food, 48% of the study population knew of other uses for the *A. heterophyllus* plant; 38.6% knew that its wood is used for heating, 8.8% knew that its wood is used for formwork, but only 1.8% knew of its use in branding. Seventy‐six percent of those surveyed harvested jackfruit in the rainy season and only 14% harvested it in the dry season. Of these, 61.7% used the picking technique and 35% used the plucking technique. After harvesting, more than half (55.5%) consumed it directly, but 32.1% kept it at room temperature and 10.7% in a cool place.

### 3.2 Physicochemical composition of fruits

The physicochemical composition of the pulp and seeds of the fruit of *A. heterophyllus* is presented in Table 1.

**TABLE 1:** Physicochemical composition of the pulp and seeds of the fruit of *Artocarpus heterophyllus*.

Physicochemical parameters (%)	Moisture content	Dry matter content	Ash content	pH	Titratable acidity (meq)
Pulp of *A. heterophyllus*	89.85 ± 0.49^a^	10.15 ± 0.21^b^	4.35 ± 0.07^a^	5 ± 0^b^	12.88 ± 1.58^a^
Boiled seeds of *A. heterophyllus*	60.075 ± 0.12^b^	39.93 ± 0.11^a^	3.45 ± 0.07^b^	6 ± 0^a^	5.62 ± 1.48^b^

*Note*: Means ± standard deviations with different letters in the same column are significantly different at the 5% level.

The pulp has significantly higher water (89.85% ± 0.49) and ash (4.35% ± 0.07) contents than the seeds (60.075% ± 0.12 and 3.45% ± 0.07, respectively, for moisture and ash contents) with P˂ 0.05. However, it had a significantly lower pH (5 ± 0) than boiled seeds (6 ± 0) and significantly higher titratable acidity (12.88 meq ± 1.58) than boiled seeds (5.62 meq ± 1.48) with *p* ˂ .05.

### 3.3 Macronutrient composition

The macronutrient composition of the pulp and seeds of the fruit of *A. heterophyllus* is presented in Table 2.

**TABLE 2:** Macronutrient composition of pulp and boiled seeds of *Artocarpus heterophyllus* fruit.

Macronutrient content (%)	Carbohydrates	Proteins	Lipids	Fibers
Pulp of *A. heterophyllu*s	54.39 ± 0.47^a^	18.35 ± 0.04^b^	6.68 ± 0,07^a^	9.88 ± 0^b^
Boiled seeds of *A. heterophyllus*	49.01 ± 0.43^b^	21.66 ± 0.31^a^	5.47 ± 0.07^b^	14.26 ± 0^a^

*Note*: Means ± standard deviations with different letters in the same column are significantly different at the 5% level.

From this table, it can be seen that the pulp has a carbohydrate content (54.39% ± 0.47) significantly higher than that of boiled seeds (49.01% ± 0.43), a protein content (18.35% ± 0.04) significantly lower than that of boiled seeds (21.66% ± 0.31), lipid content (6.68% ± 0.07) significantly higher than that of boiled seeds (5.47% ± 0.07), and fiber content (9.88% ± 0) significantly lower than that of boiled seeds (14.26% ± 0) with *p* ˂ .05.

### 3.4 Energy value of the fruit

The energy value of jackfruit pulp and seeds is presented in Table 3. From this table, we can be seen that the energy values of the different samples vary between 332.23 and 351.08 Kcal/100 g DM for boiled seeds and pulp, respectively.

**TABLE 3:** Energy value of jackfruit pulp and seeds.

Samples	Energy values kcal/100 g DM
Pulp	351.08
Boiled seeds	332.23

Abbreviation: DM, Dried Matter.

### 3.5 Mineral composition of the pulp and seeds

Table 4 shows the mineral contents (K, Na, Ca, Mg, and P) of the pulp and seeds of the fruit of *A. heterophyllus*. From this table, it is found that the sodium content of pulp (69.53 mg ± 0) is significantly higher than that of boiled seeds (57.6 mg ± 0) while the phosphorus content of boiled seeds (101.51 mg ± 4.12) is significantly higher than that of pulp (43.22 mg ± 0.13) with *p* ˂ .05. However, the potassium content of the pulp (848.75 mg ± 10.34) is higher than that of the boiled seeds (721.99 mg ± 9.57), the calcium content of the boiled seeds (132 mg ± 9.42) is higher than that of the pulp (84 mg ± 5.65), and the magnesium content of boiled seeds (43.73 mg ± 9.16) is higher than that of pulp (41.31 mg ± 5.72) although no significant difference was observed (*p* > .05).

**TABLE 4:** Mineral contents of pulp and seeds of *Artocarpus heterophyllus* fruit.

Samples	Minerals (mg)				
	K	Na	Ca	Mg	P
Pulp of *A. heterophyllus*	848.75 ± 10.34^a^	69.53 ± 0^a^	84 ± 5.65^a^	41.31 ± 5.72^a^	43.22 ± 0.13^b^
Boiled seeds of *A. heterophyllus*	721.99 ± 9.57^a^	57.6 ± 0^b^	132 ± 9.42^a^	43.73 ± 9.12^a^	101.51 ± 4.02^a^

*Note*: Means ± standard deviations with different letters in the same column are significantly different at the 5% level.

### 3.6 Antinutritional composition

Table 5 presents the antinutritional composition in the pulp and seeds of the fruit of *A. heterophyllus*. From this table, it appears that oxalates are the major antinutritional of jackfruit, the boiled seeds have a higher content of oxalates (40.5 mg ± 0) than the pulp (39.08 mg ± 0.795) even if there is no significant difference (*p* > .05). Likewise, they present contents of condensed tannins (3.54 mg ± 0.22), hydrolyzable tannins (9.87 mg ± 0.76), and saponins (0.1 mg ± 0) significantly higher than those of the pulp (2.49 mg ± 0.11, 4.13 mg ± 0.02, and 0.02 mg ± 0 for condensed tannins, hydrolyzable tannins, and saponins, respectively) with *p* ˂ .05. However, the pulp showed significantly higher phytate content (12.21 mg ± 0) than the boiled seeds (6.1 mg ± 0) with *p* ˂ .05.

**TABLE 5:** Composition of antinutritional in the pulp and seeds of the fruit of *A. heterophyllus*

Antinutritional content (mg/100 g DM)	Condensed tannins	Hydrolyzed tannins	Oxalates	Phytates	Saponins
Pulp of *A. heterophyllus*	2.49 ± 0.11^b^	4.13 ± 0.02^b^	39.08 ± 0.795^a^	12.21 ± 0^a^	0.02 ± 0^b^
Boiled seeds of *A. heterophyllus*	3.54 ± 0.22^a^	9.87 ± 0.76^a^	40.5 ± 0^a^	6.1 ± 0^b^	0.1 ± 0^a^

*Note*: Means ± standard deviations with different letters in the same column are significantly different at the 5% level.

Abbreviation: DM, Dried Matter.

### 3.7 Antioxidant activity

#### 3.7.1 Total phenolic content

The total phenolic contents of boiled jackfruit pulp and seeds are presented in Table 6. The calibration curve established gave a straight line of the equation: *y* = 0.039*x*; with a coefficient of determination *R* ^2^ = .961. The total phenolic contents of the different extracts (aqueous, ethanolic, and hydroethanolic) of boiled seeds and jackfruit pulp are between 17.94–27.65 and 12.46–13.82 mg EAG/g extract, respectively. Furthermore, aqueous and ethanolic solvents are the best extraction solvents for seeds and pulp, respectively.

**TABLE 6:** Total phenolic contents of the different extracts of the pulp and boiled seeds of jackfruit.

Various extracts	Total phenolic content of boiled seeds	Total phenolic content of the pulp
Aqueous extract	27.65 ± 1.72^a^	12.46 ± 0.25^b^
Ethanolic extract	17.94 ± 5.14^a^	13.82 ± 0.65^b^
Hydroethanolic extract	25.41 ± 3.94^a^	12.92 ± 3.49^b^

*Note*: Means ± standard deviations with different letters in the same column are significantly different at the 5% level.

#### 3.7.2 Flavonoid content

Table 7 shows the flavonoid contents of the different extracts of jackfruit pulp and seeds. The reference compound used for the establishment of the calibration curve is catechin. This curve gave a straight line of equation *y* = 0.3191*x* with a coefficient of determination *R* ^2^ = .991. The flavonoid contents of the different extracts (aqueous, ethanolic, and hydroethanolic) of boiled seeds and jackfruit pulp are between 8.85 and 10.60 mg ECAT/g extract for boiled seeds and between 6.12 and 7.01 mg ECAT/g extract for pulp. On the other hand, hydroethanolic (10.60 mg ECAT/g extract) and aqueous (7.01 mg ECAT/g extract) solvents are the best extraction solvents for boiled seeds and pulp, respectively.

**TABLE 7:** Total flavonoid content of the different extracts from the pulp and boiled seeds of jackfruit.

Various extracts	Total flavonoid content of boiled seeds	Total flavonoid content of pulp
Aqueous extract	10.55 ± 1.05^a^	7.01 ± 0.32^b^
Ethanolic extract	8.85 ± 1.77^a^	6.86 ± 0.68^b^
Hydroethanolic extract	10.60 ± 1.04^a^	6.12 ± 0.30^b^

*Note*: Means ± standard deviations with different letters in the same column are significantly different at the 5% level.

#### 3.7.3 DDPH test

**FIGURE 4:**
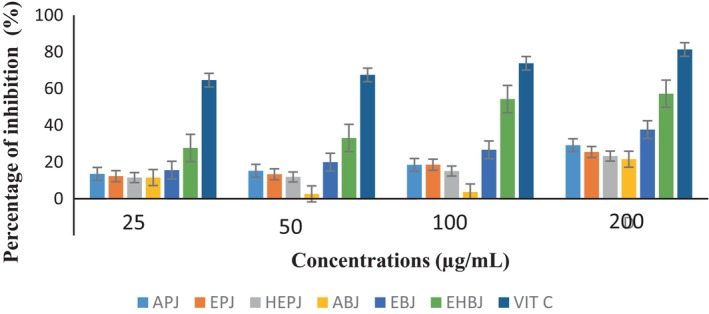
Evolution of the antifree radical activity of the different extracts of the fruit of *Artocarpus heterophyllus* at different concentrations compared to that of vitamin C. ABJ, aqueous extract of boiled jackfruit seeds; APJ, aqueous extract of jackfruit pulp; EBJ, ethanolic extract of boiled jackfruit seeds; EPJ, ethanolic extract of jackfruit pulp; HEBJ, hydroethanolic extract of boiled jackfruit seeds.; HEPJ, hydroethanolic extract of jackfruit pulp.

The evolution of the antiradical activity of the different extracts of the fruit of *A. heterophyllus* and vitamin C according to different concentrations is presented in Figure 4. It is observed that the antiradical activity varies according to the extracts and increases with the concentration. It is observed that by varying the concentrations from 25 to 100 μg/mL, the inhibition percentages of the different extracts of the pulp are similar and lower than that of vitamin C. But at 200 μg/mL, the aqueous extract shows the best activity. For boiled seeds, the inhibition percentage of the hydroethanolic extract is higher than those of the different extracts and lower than that of vitamin C whatever the concentration used (25–200 μg/mL). The aqueous extract shows the best antifree radical activity for the pulp while the hydroethanolic extract shows the best antifree radical activity for the boiled seeds.

The EC_50_ of the different extracts is presented in Table 8. It is found that the EC_50_ of the different extracts varies between 107.15 and 8972.55 μg/mL for boiled seeds and between 5075.71 and 36506.27 μg/mL for pulp. According to the classification of Souri et al. (2008), “For EC_50_ < 20 μg/mL, the antioxidant activity is high; for 20 μg/mL < EC_50_ < 75 μg/mL, the antioxidant activity is moderate; and for EC_50_ > 75 μg/mL, the activity is low;” the antioxidant activity of boiled seeds and pulp is low. However, the hydroethanolic extract of boiled seeds with the lowest EC_50_ has the best activity.

**TABLE 8:** EC_50_ of different extracts of boiled seeds and jackfruit pulp.

Various extracts	EC_50_ (μg/mL) of boiled seeds	EC_50_ (μEC_50_; μg/mL) of the pulp
Aqueous extract	8972.55 ± 3.5	5075.71 ± 2.5
Ethanolic extract	758.57 ± 1.9	11054.96 ± 4.3
Hydroethanolic extract	107.15 ± 1.7	36506.27 ± 7.5

#### 3.7.4 Measurement of hydroxyl radical inhibition

**FIGURE 5:**
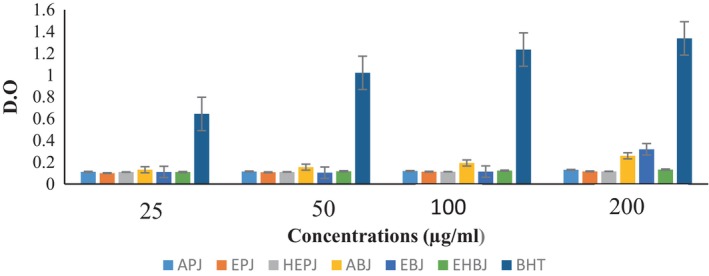
Evolution of the inhibition capacity of the hydroxyl radical of the different extracts of seeds and pulp of jackfruit. ABJ, aqueous extract of boiled jackfruit seeds; APJ, aqueous extract of jackfruit pulp; EBJ, ethanolic extract of boiled jackfruit seeds; EPJ, ethanolic extract of jackfruit pulp; HEBJ, hydroethanolic extract of boiled jackfruit seeds; HEPJ, hydroethanolic extract of jackfruit pulp.

Figure 5 shows the evolution of the hydroxyl radical inhibition capacity of the different jackfruit seed and pulp extracts. By varying the concentrations (25–200 μg /mL), the hydroxyl radical inhibition capacity of the different extracts of the pulp is similar, does not vary, and is largely lower than that of BHT. However, by varying the concentrations (25–200 μg /mL), the hydroxyl radical inhibition capacity of the different extracts of the boiled seeds does not vary up to 200 μg/mL; but from 200 μg /mL onwards it increases slightly in the aqueous and ethanolic extracts although it remains lower than that of BHT.

The ethanolic extract at 200 μg/mL has the best capacity for boiled seeds.

#### 3.7.5 Evaluation of the reducing power of ferric ions

**FIGURE 6:**
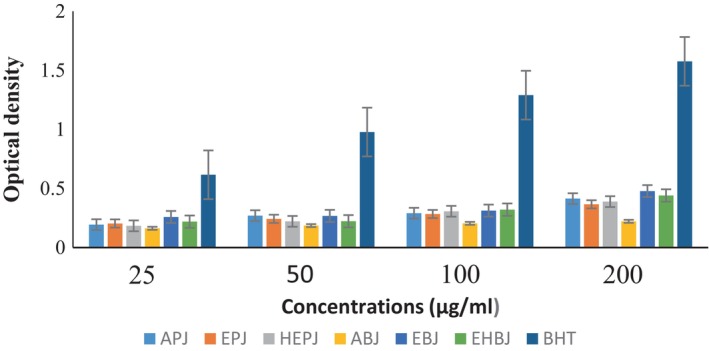
Evolution of the reducing power of the different extracts from the seeds and the pulp of jackfruit compared to that of BTH. ABJ, aqueous extract of boiled jackfruit seeds; APJ, aqueous extract of jackfruit pulp; EBJ, ethanolic extract of boiled jackfruit seeds; EPJ, ethanolic extract of jackfruit pulp; HEBJ, hydroethanolic extract of boiled jackfruit seeds.; HEPJ, hydroethanolic extract of jackfruit pulp.

The evolution of the reducing power of ferric ions of the different extracts of *A. heterophyllus* fruit at different concentrations compared to that of BHT is presented in Figure 6. It was found that by varying the concentrations from 25 to 100 μg/mL, the reducing powers of the different pulp extracts are all similar, but at 200 μg/mL, the aqueous extract shows the best power. For boiled seeds, the reducing power of the ethanolic extract is higher than those of the different extracts whatever the concentration used (25–200 μg/mL). However, it should be noted that the powers of the different extracts are all lower than that of BHT.

## 4 DISCUSSION

In the survey conducted, only 25% of the population interviewed knew jackfruit, which makes it a little‐known fruit in the eastern region of Cameroon. Regarding the consumption of jackfruit, the study showed that 98% of the people who knew jackfruit consumed it, which means that people who knew jackfruit consumed it. Regarding the different forms of consumption of jackfruit, the study revealed that the pulp was consumed more than the seeds. The seeds were eaten in a burnt or boiled state, which oriented the study toward boiled seeds. In terms of the nutritional values of jackfruit, the study revealed that the participants did not know the nutritional benefits of jackfruit or their interest in this work.

The moisture content provides information on the shelf life of the food. The water content of the obtained pulp (89.85%) is higher than those of Goswami and Chacrabati (2016) who showed that in Bangladesh, the water contents of pulps of different jackfruit varieties were 80.95%–82.22%. It is also higher than that obtained by Ouatara (2015) on mango pulp in Burkina Faso (84.53%–85.91%). However, the moisture content of boiled seeds (60.075%) is higher than that obtained by Abedin et al. (2012) in Bangladesh (51.6%–57.7%). These differences can be attributed to the cooking of seeds, climatic conditions, the nature of the soil, and the maturity of the fruit. This important water content of jackfruit pulp is a limitation for the storage time of this fruit, making it a perishable commodity. However, this high water content can also be beneficial in the production of juice in quantity. To preserve the jackfruit for a long period of time, techniques such as drying, keeping fresh, and processing into food products (jams, syrup, and chic) could be used.

The pH and titratable acidity provide information on the acidity level and organoleptic properties of food. The pulp pH value of 5 agrees with work conducted by Galvez and Dizon (2017) in the Philippines that showed jackfruit pulp pH of 4.8–5.50. This result is lower than the 4.17 found by Ouatara (2015) in Burkina Faso on mango pulp. Similarly, the pH result of boiled jackfruit seeds (6) agrees with the work conducted by Ocloo et al. (2010) in Ghana who showed that jackfruit seeds had a pH of 5.78.

Proteins play a major role in our body because of their numerous functions. The highest protein content was observed in boiled seeds (21.66%), this result is largely superior to those obtained by Thatsanasuwan et al. (2023) who worked on jackfruit seeds in Thailand and obtained protein contents of 11.83%. However, regarding jackfruit pulp, the protein content obtained was 18.35%; this result is higher than those obtained by Rajneesh and Singh (2020) in India on jackfruit pulp (1.4%–2.3%). It is also higher than those found by Haq (2006) on jackfruit pulp in Bangladesh (1.2%–1.9%). These differences can be attributed to several factors such as the genetic makeup of the plant, climatic conditions, fruit maturity stage, soil type, and sample preparation (Morris et al., 2004). The analyzed samples have a significant protein potential that could be exploited in human nutrition, especially in the production of baby food and livestock feed.

Carbohydrates are the most macronutrients in the human diet, with an intake between 45% and 50% (ANSES, 2011). The carbohydrate content of our jackfruit pulp (54.39%) is higher than that of Chrips et al. (2008) who worked on jackfruit pulp in India and obtained carbohydrate contents of 23.8%–31.2%, respectively. It is also higher than that (8.41%–11.20%) found by Ouatara (2015) on mango pulp in Burkina Faso. On the other hand, the carbohydrate content obtained with the boiled seeds studied was 49.01%. The high carbohydrate content of the jackfruit samples studied makes them a significant source of sugars that provide calories necessary for the proper functioning of the body. They give the fruit a sweet taste and pleasant aroma. This high carbohydrate content could facilitate the transformation of jackfruit into food products such as jams, syrups, chips, and juices.

The energy values of jackfruit pulp from Bertoua (351.08 Kcal/100 g) and jackfruit seeds from Bertoua (332.23 Kcal/100 g) are significantly higher than those of some exotic fruits such as avocado (155 Kcal/100 g), mango (64.4 Kcal/100 g), guava (68 Kcal), and orange (47 Kcal/100 g; Bemmo et al., 2023). Hence, jackfruit pulp and seeds from Bertoua could ensure the energy security of the populations of Eastern Cameroon.

Fibers are elements that participate in the proper functioning of intestinal transit. The highest fiber content was found in boiled jackfruit seeds (14.26%). This result is higher than that obtained by Ocloo et al. (2010) who worked on jackfruit seeds in Ghana and obtained a value of 3.19%. Regarding the pulp, the fiber content obtained was 9.88 which is higher than that of Haq (2006) who showed that jackfruit pulps in Bangladesh had fiber contents between 1% and 1.5%. This result is also higher than that (1.87%–2.77%) found by Traore (2020) on pineapple pulp in Burkina Faso. These high fiber contents make jackfruit harvested in Bertoua, Cameroon an essential food in the prevention of constipation, colon cancer, diabetes, and cardiovascular diseases (Bemmo et al., 2023).

Among the minerals analyzed, potassium was the mineral with the highest content in the pulp and boiled seeds of *A. heterophyllus* fruit (848.75 mg/100 g and 721.99 mg/100 g, respectively). Indeed, potassium is a very important mineral because it allows, among other things, to maintain the acid‐basic balance, the osmotic pressure, and the conduction of nerve impulses. This high potassium content in jackfruit from Bertoua makes it an important source of potassium to prevent potassium deficiencies or for formulations. Potassium is also hypotensive and is also involved in muscle contraction (Dedehou et al., 2015) so consumption of jackfruit from Bertoua, Cameroon should be advised for people with high blood pressure.

Calcium is the second most encountered mineral in our samples. Calcium contents are 84 ± 5.65 mg/100 g for pulp and 132 ± 9.42 mg/100 g for seeds. Calcium is important for the construction and maintenance of bones, blood coagulation, transmission of nerve impulses, and formation of teeth and bones. It is also an important cofactor in enzymatic metabolic processes (Senga et al., 2013). The consumption of jackfruit pulp and seeds from Bertoua should be encouraged in growing toddlers and people suffering from osteoporosis (insufficient bone mineralization).

Phosphorus is the third most encountered mineral in our samples (43.22 mg/100 g for pulp and 101.51 mg/100 g for seeds). It helps strengthen bones and teeth. It is very essential for the fortification of children and nursing mothers (Andzouana & Mombouli, 2012).

Sodium is also a mineral found in the pulp (69.53 mg/100 g) and the seeds (57.6 mg/100 g) of the *A. heterophyllus* fruit harvested in Bertoua, Cameroon. It maintains the acid–base and osmotic balance between the cells and the interstitial liquid (Martin, 2001).

The analysis of the nutritional value of the different samples allowed us to say that jackfruit pulp and seeds from Bertoua, Cameroon are rich in macronutrients (carbohydrates, proteins, and fiber) and micronutrients (potassium, calcium, phosphorus, magnesium, and sodium).

However, when antinutrients are present in foods, they sometimes prevent the absorption of certain nutrients by several mechanisms. Thus, the content of some antinutrients has been evaluated in this study.

The antinutrients that were determined in our study were as follows: phytates, tannins, saponins, and oxalates.

Oxalates were the most common antinutrients found in our samples. A high concentration of oxalates in the diet could lead to kidney stones (Nwaogu & Emejulu, 2010). The analyses carried out showed that the boiled seeds and pulp of *A. heterophyllus* contained 40.5 and 39.08 mg oxalates/100 g, respectively. These results for jackfruit pulp from Bertoua are significantly lower than those obtained by Adetuyi et al. (2008) on papaya pulp (450 mg/100 g) in Nigeria.

Phytates prevent the absorption of iron, calcium, zinc, and magnesium. They must, therefore, be found in very small quantities in food. The analyses carried out showed that our samples of boiled seeds and pulp of *A. heterophyllus* contained 6.1 and 12.21 mg of phytates/100 g. The results of jackfruit pulp of this study are lower than those obtained by Phillippy and Wyatt (2001) and Adetuyi et al. (2008) on carrot and avocado in New Orleans and papaya in Nigeria, respectively. The results of boiled seeds are lower than those obtained by Kumar and Sinha (2018) on maize.

Tannins are substances that prevent the absorption of iron, a mineral that is very important for the proper functioning of the body. They precipitate proteins and thus reduce food intake (Atelor, 1993). The hydrolyzed and condensed tannin contents of boiled seed samples were, respectively, 9.87 mg/100 g and 3.54 mg/100 g. The hydrolyzed and condensed tannin contents of pulp samples were 4.13 mg/100 g and 2.49 mg/100 g. These results for jackfruit pulp from Bertoua are lower than those obtained by Adetuyi et al. (2008) on papaya: 21 mg/100 g hydrolyzed tannins and 62 mg/100 g condensed tannins in Nigeria.

Saponins are substances that are astringent at high concentrations and impart a bitter taste to fruits. High saponin levels have been associated with gastrointestinal enteritis manifested by diarrhea and dysentery (Datti et al., 2020). The saponin contents of the samples were 0.1 mg/100 g for boiled seeds and 0.02 mg/100 g for jackfruit pulp, respectively. These results obtained in the jackfruit pulp samples are lower than those of Umuru et al. (2007) who showed that the pulp of baobab fruit in Nigeria had a saponin content of 1.051 mg/100 g.

Given the above results, the jackfruit pulp and seeds from Bertoua contain lower antinutritional values than those of widely consumed fruits, so we can conclude that they are consumable.

The antioxidant activity of the different extracts by colorimetric determination of total phenols and flavonoids proved that the flavonoid contents of the fruits varied according to the aqueous, ethanolic, and hydroethanolic solvents. Total phenols and flavonoids are natural antioxidants. The highest content of total phenols was observed in boiled seeds (14.39 mg EAG/g extract). This result is lower than those obtained by Jagtap et al. (2010) who worked on jackfruit seeds in India and obtained a phenolic content of 27.7 mg EAG/g extract. However, the result of the phenolic content of pulp (13.82 mg EAG/g extract) is higher than those found by Soong and Barlow (2004); Jagtap et al. (2010) who showed that jackfruit pulps in Singapore and India had total phenolic contents of 0.9 and 0.46 mg EAG/g extract, respectively. This variation could be due to the complex nature of this group of compounds, the extraction, or the analysis method used (Kalt et al., 2001). It has been shown that the phenolic composition of the plant is influenced by intrinsic factors such as species and variety or by extrinsic factors such as environment and agronomy (Barberan & Espin, 2001). Regular consumption of jackfruit pulp and seeds from Bertoua could prevent oxidative stress‐related disorders such as degenerative diseases.

Epidemiological and clinical studies have highlighted the potential role of flavonoids in reducing the risk factors for cardiovascular diseases, osteoporosis, and lung cancer (Lampila et al., 2009). In the analyses, the best solvent for the extraction of flavonoids in the pulp was water, this result agrees with the work conducted by Jagtap et al. (2010) on jackfruit pulp in India. On the other hand, the flavonoid content obtained in the pulp samples (7.01 mg EAG/g extract) is higher than that found by the same author in jackfruit pulp in India (1.2 mg RE/extract). The concentration of flavonoids in jackfruit is an asset for health since flavonoids, by their function, protect blood vessels from cholesterol damage and reduce oxidative stress (because they are antioxidants).

The antioxidant activity was also evaluated by three tests: the 1,1‐diphenyl‐2‐picrylhydrazyl radical (DPPH) test, the FRAP test, and the ability to reduce the hydroxyl radical. Regarding the DPPH test, all the pulp extracts were able to reduce the DPPH radical; this result agrees with the studies conducted by Jagtap et al. (2010) who worked on jackfruit pulps in India. However, the EC_50_ of the pulp sample from Bertoua (5.075–36.506 mg/mL) is higher than the one found by the same author in India on jackfruit pulp (0.4–0.7 mg/mL).

The antioxidant‐reducing power of ferric ions is the simplest, quickest, and cheapest method for routine analysis. The analyses performed showed that all extracts (pulp and boiled seeds) have ferric ion‐reducing power; however, these reducing powers are lower than that of BHT. These results are contrary to those obtained by Jagtap et al. (2010) who demonstrated that at low concentrations (1–3 mg/mL) all extracts of jackfruit pulp in India had higher ferric ion‐reducing powers than vitamin C. These differences can be attributed to the method used.

Jackfruit pulp and seeds from Bertoua, Cameroon, have excellent natural antioxidants that could be used to fight oxidative stress.

## 5 CONCLUSION

This study revealed that jackfruit (*Artocarpus heterophyllus)* pulp and seeds from Bertoua, Eastern Region, Cameroon, have important nutritional value and antioxidant properties exploitable in human nutrition. Hence, they can be used to improve the nutritional status of the populations in this region where the malnutrition level is still high. According to its high energy value (more than 300 Kcal/100 g), jackfruit from Bertoua, Cameroon, could be used in infant nutrition. The consumption of this fruit should be encouraged. For further studies, we intend to evaluate the vitamin contents of jackfruit pulp from Bertoua and identify the phenolic compounds present in this fruit.

## AUTHOR CONTRIBUTIONS

**Ulrich Landry Kamdem Bemmo:** Conceptualization (lead); investigation (equal); methodology (equal); writing – original draft (equal); writing – review and editing (equal). **Jean Marcel Bindzi:** Data curation (equal); formal analysis (equal); writing – original draft (equal); writing – review and editing (equal). **Pamela Regine Tayou Kamseu:** Investigation (equal); methodology (equal); writing – original draft (equal); writing – review and editing (equal). **Serge Cyrille Houketchang Ndomou:** Writing – original draft (equal); writing – review and editing (equal). **Stephano Tene Tambo:** Methodology (equal); writing – review and editing (equal). **François Ngoufack Zambou:** Supervision (lead); validation (equal); writing – original draft (equal); writing – review and editing (equal).

## ACKNOWLEDGEMENTS

The authors thank Professor Zambou François who kindly allowed this work to take place in his Research Unit.

## CONFLICT OF INTEREST STATEMENT

The authors confirm that they have no conflicts of interest concerning the work described in this manuscript.

## ETHICS STATEMENT

All panelists enrolled in this study provided written informed consent. Also, they were informed that they can withdraw from the evaluation at any time without giving a reason.

## Data Availability

There are no primary data associated with this manuscript.

## References

[fsn33437-bib-0001] Abedin, M. S. , Nuruddin, M. M. , Ahmed, K. U. , & Hossain, A. (2012). Nutritive composition of locally available jackfruit seeds (*Artocarpus heterophyllus*) in Bangladesh. International Journal of Biosciences, 8, 1–7.

[fsn33437-bib-0002] Adetuyi, F. O. , Ayileye, T. A. , & Dada, I. B. O. (2008). Comparative study of quality changes in shea butter coated pawpaw *Carica papaya* fruit during storage. Pakistan Journal of Nutrition, 7(5), 658–662.

[fsn33437-bib-0003] Andzouana, M. , & Mombouli, J. (2012). Assessment of the chemical and phytochemical constituents of the leaves of a wild vegetable‐*Ochthocharis dicellandroides* (Gilg). Pakistan Journal of Nutrition, 11, 94–99. 10.3923/pjn.2012.94.99

[fsn33437-bib-0004] ANSES . (2011). Update of the recommended dietary allowances for fatty acids. Rapport d'expertise collective. Scientific edition.

[fsn33437-bib-0005] AOAC . (2005). Official methods of analysis (18th ed.). Association of Official Analytical Chemists.

[fsn33437-bib-0006] AOAC (Association of Official Analytical Chemists) . (1990). Official methods of analysis (pp. 80, 912, 915, 917, 918). Association of Official Analytical Chemists, Inc.

[fsn33437-bib-0007] Atelor, V. A. (1993). Allelochemicals in plant food and feeding stuff: 1. Nutritional, biochemical, and physiopathological aspects in animal production. Toxicology, 35, 57–67 PMID: 8434459. 8434459

[fsn33437-bib-0008] Barberan, T. F. , & Espin, J. C. (2001). Phenolic compounds and related enzymes as determinants of the quality of fruits and vegetables. Journal of Science Food and Agriculture, 81, 853–876. 10.1002/jsfa.885

[fsn33437-bib-0009] Bemmo, K. U. L. , Mvongo, C. , Bindzi, J. M. , Ekwelle, M. W. N. , & Zambou, N. F. (2023). Contribution to the valorization of *Myrianthus arboreus* fruits pulp from Cameroon: Physico‐chemical characterization and nutritional value. Mesurement: Food, 10(2023), 100083. 10.1016/j.meafoo.2023.100083

[fsn33437-bib-0010] Chrips, N. R. , Balasingh, G. R. , & Kingston, C. (2008). Nutrient constituents of neglected varieties of Artocarpus heterophyllus Lam. From Kanyakumari district, South India. Journal of Basic and Applied Biology, 2(3&4), 36–37.

[fsn33437-bib-0011] Datti, Y. , Tijjani, Y. A. , Koki, I. B. , Ali, U. L. , Labaran, M. , Ahmad, U. U. , & Tasi'u, N. (2020). Phytochemical composition of desert date kernel (*Balanites aegyptiaca*) and the physical and chemical characteristics of its oil. GSC Biological and Pharmaceutical Sciences, 11(3), 197–207. 10.30574/gscbps.2020.11.3.0166

[fsn33437-bib-0012] Dedehou, E. S. C. A. , Dossou, J. , & Soumanou, M. M. (2015). Diagnostic study of cashew apple juice processing technologies in Benin. International Journal of Biological and Chemical Science, 9, 371–387. 10.4314/ijbcs.v9i1.32

[fsn33437-bib-0013] Dif, M. M. , Benchiha, H. , Mehdadi, Z. , Benali‐Toumi, F. , Benyahia, M. , & Bouterfas, K. (2015). Quantitative study of polyphenols in different organs of *Payer rhoes* L. Phytothérapie, 13, 314–319. 10.1007/s10298-015-0976-5

[fsn33437-bib-0014] Galvez, L. , & Dizon, E. (2017). Physico‐chemical and functional proprieties of fresh jackfruit (*Artocarpus heterophyllus* Lam) varieties in eastern Visayas Philippines. Annals of Tropical Research, 39, 100–106. 10.32945/atr3929.2017

[fsn33437-bib-0015] Goswami, C. , & Chacrabati, R. (2016). Chapter 14‐jackfruit (*Artocarpus heterophyllus*). In M. S. J. Simmonds & V. R. Preedy (Eds.), Nutritional composition of fruit cultivars (Vol. 2016, pp. 317–335). Academic Press.

[fsn33437-bib-0016] Haq, N. (2006). Jackfruit (*Artocarpus heterophyllus*). In J. T. Williams , R. W. Smith , & Z. Dunsiger (Eds.), Tropical fruit trees. Southampton Centre for Underutilised Crops, University of Southampton.

[fsn33437-bib-0017] Ijarotimi, S. O. , & Keshinro, O. O. (2013). Determination of nutrient composition and protein quality of potential complementary foods formulated from the combination of fermented popcorn, African locust, and Bambara groundnut seed flour. Polish Journal of Food and Nutrition Sciences, 63(3), 155–166. 10.2478/v10222-012-0079-z

[fsn33437-bib-0018] Institut National de la Statistique (INS), ICF . (2020). Enquête Démographique et de Santé du Cameroun 2018. INS et ICF.

[fsn33437-bib-0019] Jagtap, U. B. , Panaskar, S. N. , & Bapat, A. V. A. (2010). Evaluation of antioxidant capacity and phenol content in jackfruit (*Artocarpus heterophyllus* Lam.) fruit pulp. Plant Foods for Human Nutrition, 65, 99–104. 10.1007/s1130-010-0155-7 20198442

[fsn33437-bib-0020] Jahromi, S. G. (2019). Extraction techniques of phenolic compounds from plants. In C. S. Nunes , & V. Kumar (Eds.), From the edited volume: Plant physiological aspects of phenolic compounds (pp. 1–18). Intech Open. 10.5772/intechopen.84705

[fsn33437-bib-0021] Kalt, W. , Ryan, D. , Duy, J. C. , Prior, R. L. , Ehlenfeldt, M. K. , & Kloet, S. P. V. (2001). Interspecific variation in anthocyanins, phenolics, and antioxidant capacity among genotypes of high bush and low bush blueberries (Vaccinium section *Cyanococcus* spp.). Journal of Agricultural and Food Chemistry, 49, 4761–4767. 10.1021/jf010653e 11600018

[fsn33437-bib-0022] Kassegn, H. H. (2018). Determination of proximate composition and bioactive compounds of the Abyssinian purple wheat. Cogent Food & Agriculture, 4(1), 1421415. 10.1080/23311932.2017.142141

[fsn33437-bib-0023] Kaur, J. , Singha, Z. S. , Mazharb, H. M. S. , M. S. , Hasana, M. , & Woodward, A. (2023). Insights into phytonutrient profile and postharvest quality management of jackfruit: A review. Critical Reviews in Food Science and Nutrition, 1–27. 10.1080/10408398.2023.2174947 36789587

[fsn33437-bib-0024] Kone, H. S. (2018). Caractérisation Biochimique De La Pulpe des Fruits Du Prunier Noir (*Vitex doniana*) De La Côte D'ivoire. European Scientific Journal, 14, 1857–7881. 10.19044/esj.2018.v14n3p252

[fsn33437-bib-0025] Kumar, V. , & Sinha, A. K. (2018). Chapter 3‐General aspects of phytases. In C. S. Nunes , & V. Kumar (Eds.), Enzymes in human and animal nutrition (pp. 53–72). Academic Press.

[fsn33437-bib-0026] Kushwaha, R. , Ayushi, G. , Singh, V. , Kaur, S. , Puranik, V. , & Kaur, D. (2023). Jackfruit seed flour‐based waffle ice cream cone: Optimization of ingredient levels using response surface methodology. Heliyon, 9(2023), e13140. doi:10.1016/j.heliyon.2023.e13140 36793960 PMC9922971

[fsn33437-bib-0027] Lampila, P. , Lieshout, M. , Gremmen, B. , & Lahteenmaki, L. (2009). Consumer attitudes towards enhanced flavonoid content in fruit. Food Research International, 42, 122–129. 10.1016/j.foodres.2008.09.002

[fsn33437-bib-0028] Martin, A. (2001). The “apports nutritionnels conseillés (ANC)” for the French population. Reproduction Nutrition Development, 41(2), 119–128. 10.1051/rnd:2001100 11434516

[fsn33437-bib-0029] Mengue, A. S. (2004). *The Eastern Province of Cameroon: A study of human geography*. Doctoral thesis in geography from the University of Bordeau 3, France.

[fsn33437-bib-0030] Mensor, L. , Menezez, F. , Leitao, G. , Reis, A. , Dos Santos, T. , Coube, C. , & Leitao, S. (2001). Screening of Brazilian plant extracts for antioxidant activity by the use of the DPPH free radical method. Phytotherapy Research, 15, 127–130. 10.1002/ptr.687 11268111

[fsn33437-bib-0031] Morris, A. , Barnett, A. , & Burrows, O. (2004). Effect of processing on the nutrient content of foods. Cajarticles, 37, 160–164.

[fsn33437-bib-0032] Nagulendran, K. , Velavan, S. , Mahesh, R. , & Haseena, B. (2007). In vitro antioxidant activity and total polyphenolic content of *Cyperusrotundus rhizomes* . Journal of Chemistry, 4, 440–449. 10.1155/2007/903496

[fsn33437-bib-0033] Nguefack, F. , Akazong, A. C. , Keugoung, B. , Kamgaing, N. , & Dongmo, R. (2015). Management of severe acute malnutrition in children with local alternative preparations to F‐75 and F‐100: Results and challenges. Pan African Medical Journal, 21, 329. 10.11604/pamj.2015.21.329.6632 26587175 PMC4633737

[fsn33437-bib-0034] Nwaogu, L. A. , & Emejulu, A. A. (2010). Evaluation of the toxicity of cyanogens in a commonly consumed Nigeria legume pigeon pea (*Cajanus cajan*) seed and its biochemical effects in rabbits. International Journal of Biological and Chemical Sciences, 4(5), 1435–1441.

[fsn33437-bib-0035] Ocloo, F. C. , Bansa, D. , Boatin, R. , Adam, T. , & Agbemavor, W. (2010). Physicochemical, functional and pasting characteristics of flour produced from jackfruits (*Artocarpus heterophyllus*) seeds. Agriculture and Biology of North America, 1, 903–908. 10.5251/abjna.2010.1.5.903.908

[fsn33437-bib-0036] Ouatara, M. (2015). Caractérisation physico‐chimique de deux variétés locales et d'une variété améliorée de mangue au Burkina Faso. Licence professionnelle en genie biologique de l'Institut de Recherches en sciences appliquées et technologie (IRSAT) (p. 45). FASO.

[fsn33437-bib-0037] Phillippy, B. Q. , & Wyatt, C. J. (2001). Degradation of phytates in foods by phytases in fruit and vegetables extracts. Food Chemistry and Toxicology, 66, 535–539. 10.1111/j.1365-2621.2001.tb04598.x

[fsn33437-bib-0038] Rajneesh, S. , & Singh, A. (2020). Jackfruit (*Artocarpus heterophyllus* Lam) biggest fruit with high nutritional and pharmacological values: A review. International Journal of Current Microbiology and Applied Sciences, 9(8), 764–774. 10.20546/ijcmas.2020.908.08

[fsn33437-bib-0039] Rechek, H. , Haouat, A. , Hamaidia, K. , Allal, H. , Boudiar, T. , Pinto, D. C. G. A. , Cardoso, S. M. , Bensouici, C. , Soltani, N. , & Silva, A. M. S. (2021). Chemical composition and antioxidant, anti‐inflammatory, and enzyme inhibitory activities of an endemic species from southern Algeria: *Warionia saharae* . Molecules, 26, 5257. 10.3390/molecules26175257 34500690 PMC8434534

[fsn33437-bib-0040] Sadler, G. D. , & Murphy, P. A. (2010). pH and Titratable acidity. In Food analysis. Food analysis. Springer. 10.1007/978-1-4419-1478-1_13

[fsn33437-bib-0041] Senga, Y. , Yoshioka, K. , Kameshita, I. , & Sueyoshi, N. (2013). Expression and gene knockdown of zebrafish Ca^2+^/calmodulin‐dependent protein kinase Idelta‐LL. Archives of Biochemistry and Biophysics, 540(1–2), 41–52. 10.1016/j.abb.2013.09.016 24099663

[fsn33437-bib-0042] Soong, Y. Y. , & Barlow, P. J. (2004). Antioxidant activity and phenolic content of selected fruit seeds. Food Chemistry, 88, 411–417. 10.1016/j.foodchem.2004.02.003

[fsn33437-bib-0555] Souri, E. , Amin, G. , Farsam, H. , & Barazandeh, T. M. (2008). Screening of antioxidant activity and phenolic content of 24 medicinal plant extracts. DARU, 16(2), 83–87.

[fsn33437-bib-0043] Thatsanasuwan, N. , Duangjai, A. , Suttirak, P. , & Phanthurat, N. (2023). Proximate composition and sensory attributes of gluten‐free pasta made from jackfruit seeds. Functional Foods in Health and Disease, 13(1), 11–21. 10.31989/ffhd.v13i1.1039

[fsn33437-bib-0044] Traore, K. H. (2020). Valorisation des variétés de mangues produites au Burkina Faso: aspects biochimiques, biotechnologiques et nutritionnels (p. 170). These de doctorat de l'Université de Joseph Ki‐Zerbo. 10.13140/RG.2.2.27000.06407

[fsn33437-bib-0045] Umuru, H. A. , Adamu, D. D. , & Nadro, M. S. (2007). Levels of anti‐nutritional factors in some wild edible fruits of Northern Nigeria. African Journal of Biochtenology, 16, 1935–1938. 10.5897/AJB2007.000-2294

